# Association of perturbation of oral bacterial with incident of Alzheimer's disease: A pilot study

**DOI:** 10.1002/jcla.24483

**Published:** 2022-06-11

**Authors:** Majid Taati Moghadam, Nour Amirmozafari, Ali Mojtahedi, Babak Bakhshayesh, Aref Shariati, Faramarz Masjedian Jazi

**Affiliations:** ^1^ 440827 Student Research Committee Iran University of Medical Sciences Tehran Iran; ^2^ 440827 Department of Microbiology School of Medicine Iran University of Medical Sciences Tehran Iran; ^3^ 37554 Department of Microbiology School of Medicine Guilan University of Medical Sciences Rasht Iran; ^4^ 37554 Department of Neurology Poursina Hospital Guilan University of Medical Sciences Rasht Iran; ^5^ Molecular and medicine research center Khomein University of Medical Sciences Khomein Iran

**Keywords:** Alzheimer's disease, inflammatory cytokines, neurological disease, oral microbiome, periodontitis, qPCR

## Abstract

**Objective:**

This case‐control study was designed to compare the composition of the predominant oral bacterial microbiome in Alzheimer's disease (AD) and control group.

**Subject:**

A total of 30 adult participants (15 AD and 15 healthy individuals) were entered in this study. The composition of oral bacterial microbiome was examined by quantitative real‐time polymerase chain reaction (qPCR) using bacterial 16S rDNA gene. The levels of systemic inflammatory cytokines in both groups were assessed using enzyme‐linked immunosorbent assays (ELISA).

**Results:**

The loads of *Porphyromonas gingivalis*, *Fusobacterium nucleatum*, and *Prevotella intermedia* were significantly more abundant in the AD compared to the control group (*p *< 0.05). Although *Aggregatibacter actinomycetemcomitans* and *Streptococcus mutans* were relatively frequent in the AD group, no significance difference was observed in their copy number between two groups. Although the concentrations of IL‐1, IL‐6, and TNF‐α were higher in the AD group, there was a significant difference in their levels between the two groups (*p* < 0.05). Finally, there was a significant relationship between increased number of pathogenic bacteria in oral microbiome and higher concentration of cytokines in patient's blood.

**Conclusion:**

Our knowledge of oral microbiome and its exact association with AD is rather limited; our study showed a significant association between changes in oral microbiome bacteria, increased inflammatory cytokines, and AD.

## 1 INTRODUCTION

Alzheimer's disease (AD) is considered as a devastating neurodegenerative disease and the most common type of dementia that is associated with both central and peripheral immune dysregulations. It represents 60%–80% of dementia cases and has a multifactorial etiology.^1^, ^2^ Although it has been researched for decades, the exact cause of the disease is unknown and no definitive prevention or treatment has been proposed.^3^ Even though the causes of AD are shrouded in obscurity, aging is one of the most important risk factors. The increasing life expectancy has led to population aging,as a consequence, it is likely that AD will quadruple over the next 40 years.^4^ A number of other factors such as lifestyle, genetic signature, and environmental parameters have been contributed to AD besides aging.^5^ It is estimated that 13–14 million people in the United States are likely to suffer from AD with a treatment cost of more than 1 trillion dollars. In 2015, 47 million AD patients were diagnosed worldwide with the prediction that the number of patients would reach 75 million by 2030 and 132 million by 2050.^6^ Familial‐early‐onset and sporadic‐late‐onset are two main forms of AD based on pathogenic features which comprise around 2% and 98% of all cases, respectively. Mutations in PSEN1 (Presenilin 1), and PSEN2 (Presenilin 2) as amyloid precursor protein genes, are a reason of familial‐early‐onset form which occurs earlier in life with an extensive amyloid beta (Aβ) deposits and functional loss.^7^ On the contrary, the inheritance of Apolipoprotein ɛ4 (APOEɛ4) gene which is considered as the main cause for the late‐onset form has an indispensable environmental role in expression of APOEɛ4.^8^ Moreover, hyperphosphorylated tau (HPtau), introduced as another cerebral feature of AD, has proven to be responsible for progressive cognitive impairment. AD progression are split into three (A–C) and six (I–VI) stages according to Aβ and HPtau movements in the brain, respectively.^9^ The Aβ deposition are usually found in the isocortex of the cerebral cortex and are not equal in size and shape as well as show inter‐individual variations at early stages. Aβ plaques develop before the HPtau formation, and the development of Aβ plaques does not mean HPtau lesion will certainly develop.^10^ It is worth mentioning that in AD patients, chronic inflammation and dysfunction of the immune system occur several decades before cognitive impairment as impaired immune regulation is the first sign of AD progression.^11^ An influential factor responsible for chronic inflammation is microbiome changes, which is one of the most important issues in various diseases. Researchers are conducting various studies on the relationship between oral microbiome changes and various diseases.^12^, ^13^, ^14^, ^15^ The population of microbes which live in commensalism or symbiosis form with human is described as the “human microbiome”.^16^ Oral cavity has an intricate environment in which various microbial populations including bacteria, viruses, fungi, archaea, and protozoa live.^17^ Perturbation in the oral microbiota enhances the risk of periodontitis consequently raising Aβ peptide plaques in the brains. On the other hand, a close correlation has been declared between chronic periodontitis and intensification of systemic inflammation and reduction of cognitive functions.^18^, ^19^ In addition to neuro‐inflammation by bacterial products and Aβ deposition in the brain, there are other mechanisms for microbiome changes that progress AD, including dysbiosis in the gut microbiota, damage to oral mucosal barriers, and microbes entering brain through bloodstream.^20^ Therefore, among the essential risk factors involved in exacerbation of AD, include change in the microbiome, especially the oral microbiome. In the current study, the relationship between perturbation of inflammatory cytokines and microbiome oral bacteria was compared in Alzheimer patients and healthy individuals.

## 2 MATERIAL AND METHODS

### 2.1 Subjects and sample preparation

**FIGURE 1:**
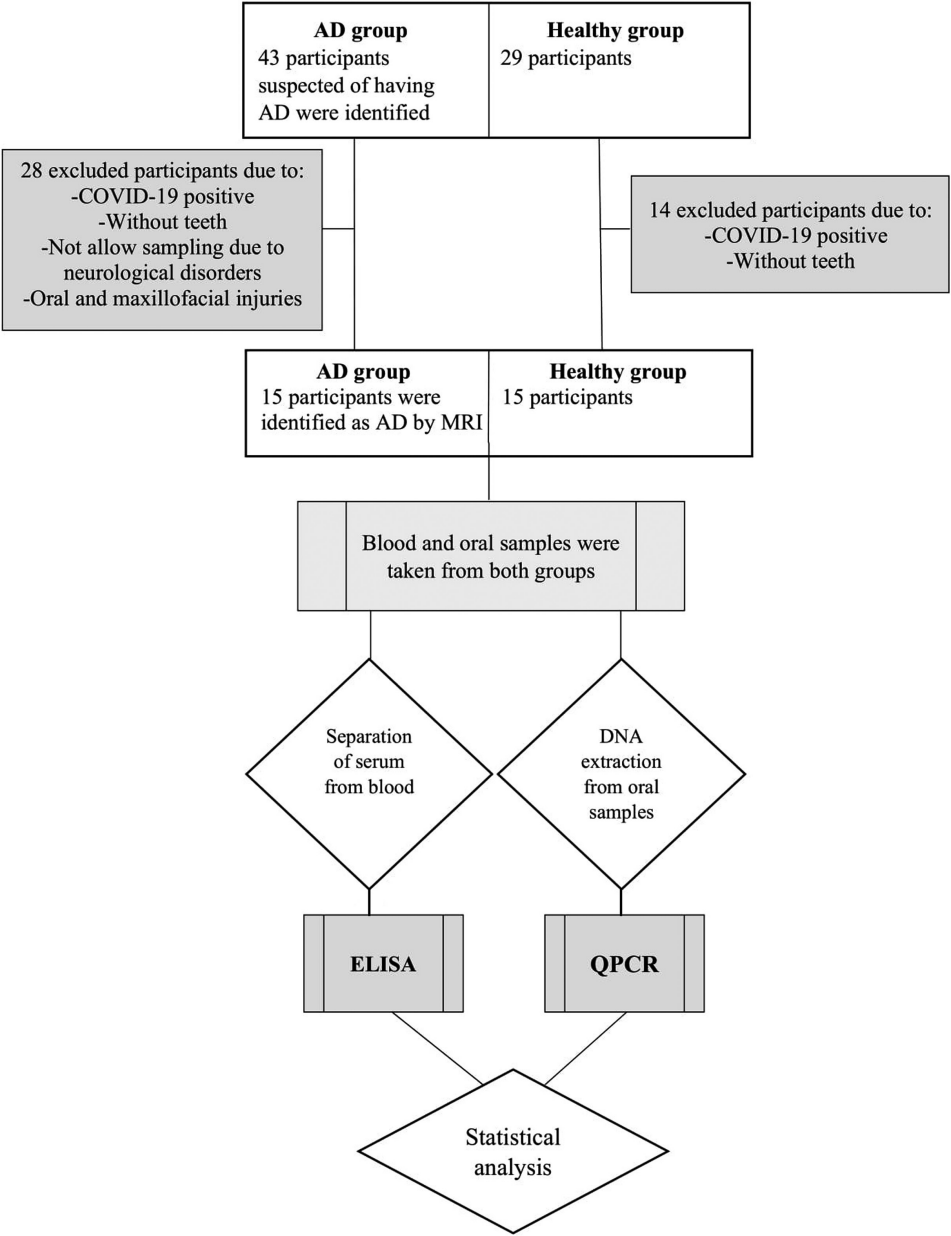
A schematic diagram of the experimental design

This cross‐sectional study was carried out during 18 months from April 2020 to October 2021 by convenient sampling method on elderly individuals hospitalized in the neurology department of Poursina hospital of Rasht city which is the provisional capital of Guilan province in Iran. All sampling methods and techniques of this study were approved by the ethics committee of Iran University of Medical Sciences (IR.IUMS.FMD.REC.1399.553). Demographic questionnaires were used for collecting data related to all variants. The mini‐mental state examination (MMSE) was used by a neurologist as one of the most common methods of measuring general cognitive function of participants in the study. Finally, brain magnetic resonance imaging (MRI) examination and blood tests were conducted for definitive diagnosis of hospitalized Alzheimer patients. The inclusion criteria for AD group included age of 60 years or above, accurate diagnosis of AD by a neurologist, and voluntary participation. The exclusion criteria included the elderly over 60 years without teeth, people despite the consent of companions who did not allow sampling due to neurological disorders, COVID‐19 positivity, and people with severe oral and maxillofacial injuries (Figure 1). Following diagnosis of AD by a neurologist and obtaining informed consent, as part of a protocol approved by the ethics committee of Iran University of Medical Sciences, 5 ml of venous peripheral blood was collected from the AD and control individuals (volunteers over 60 years of age, with teeth, without neurological disease, and COVID‐19 negative). The blood samples were then centrifuged (10 min at 2500 *g*) and sera were isolated and stored at −20°C for further examination. Oral specimens were sampled from mucosa, teeth, supra‐ and sub‐gingival spaces, tongue, and keratinized gingiva using sterile paper points according to the instructions of Santigli et al.^21^ All oral samples were fixed in phosphate‐buffered saline (PBS), then snap‐frozen and stored at −70°C until DNA extraction.

### 2.2 DNA extraction

Each paper point sample was included in 2 ml microcentrifuge tube containing 200 µl of sterile 1X PBS which was vortexed (LABINCO, L46, Netherlands) for 2 min at 300 *g* in order to release microbial cells. Thereafter, tubes were centrifuged at 1200 *g* for 10 min and supernatant was carefully removed. DNA extraction was performed from the deposits. Genomic DNA of all oral samples was extracted according to the instructions of QIAamp DNA Mini Kit (QIAGEN, Hilden, Germany). The quantity and quality of the extracted DNA were evaluated in OD 260/280 nm as well as on agarose gel (1.5%) and the confirmed DNA was stored at −20°C for further analysis.

### 2.3 Primers and probes design

TaqMan probes and primers were used for the detection of 16S rDNA gene sequence of five main oral microbiome, including *Porphyromonas gingivalis*, *Aggregatibacter actinomycetemcomitans*, *Fusobacterium nucleatum*, *Prevotella intermedia*, and *Streptococcus mutans*. Primer Express software V.3.0 (Applied Biosystems) was used to design the probes and primers, based on research conducted by Shariati et al.^22^ Table 1 exhibits the sequences and detailed characteristics of the probes and primers used in this study. Notably, 3′ and 5′ ends of all TaqMan probes were labeled with the quencher dye BHQ and reporter dye molecule FAM, respectively. In addition, to prevent the probe extension in the PCR process, the 3′ ends of each probe were phosphorylated. Finally, BLAST analysis was applied to evaluate the specificity of the sequences of primers and probes (https://blast.ncbi.nlm.nih.gov/Blast.cgi).

**TABLE 1:** The sequence of primers designed in this study to identify the composition of bacteria in the oral microbiome

Target bacteria	Primer/probe	Oligonucleotide sequence (50e30)	Product size (bp)	Reference
*Porphyromonas gingivalis*	Primer F	TCGTCGAAACGATCGAAACC	162	This study
	Primer R	GCAGAGCGGTGTAATACGTC		
	Probe	TTCGCGGTATCTTGCCGGCC		
*Aggregatibacter actinomycetemcomitans*	Primer F	CCACGCCGTTAATGTTCCAT	120	This study
	Primer R	GCCCGTAAGCCTTGCTATTC		
	Probe	AAACGCCTGTGTGCCGCGCC		
*Fusobacterium nucleatum*	Primer F	AGCTACAAGAGAAGAAAATGAAAATGG	105	This study
	Primer R	CCAACTCCTACAAATCCAGTAACC		
	Probe	TTACTTCATACCATACACGAGGATCTACTT		
*Prevotella intermedia*	Primer F	AAGACCGTGTTCAACCAACG	102	This study
	Primer R	TGTCATCACTTCCTGCTCGT		
	Probe	CTGGCGCAGGCTTACTCGCA		
*Streptococcus mutans*	Primer F	TGGAACAATCTCACCAGCCA	112	This study
	Primer R	TCGTCAGTTCTTCACCACGA		
	Probe	TGCTGCTTCCAAGGCTTGTTCCAGC		

### 2.4 Quantitative real‐time PCR (qPCR)

Specific primers and probes designed for 16SrDNA and TaqMan qPCR were used to compare the number of oral microbiome bacteria between the patient and healthy groups. The final volume of amplification reaction was 20 ml in each qPCR including a mixture of 9 µl of Universal Probe Ex Taq PCR Master Mix (Ampliqon), 0.25 µM of the probe, 0.5 µM of each forward and reverse primer, 20 ng of extracted DNA, and 5.8 ml demineralized water. The qPCR thermal‐cycling condition were performed in a Rotor‐Gene 6000 real‐time PCR cycler (Qiagen Corbett) as follows: an initial denaturation at 95°C for 15 min, annealing of 40 cycle for 15 s at 58°C, and extension at 58°C for 30 s. Non‐template control (NTC) reactions were conducted in all tests and consisted of all the substances of the amplification reaction except the template DNA. In addition, final analysis of the results was carried out on the mean values of duplicate qPCR tests on the six studied genes. Standard curves were obtained applying serially diluted four dilutions of genomic DNA according to positive control strain for each qPCR assay. In the next stage, absolute quantification was carried out via a standard curve for definition of the copy number concentration of *F*.* nucleatum* ATCC 25586, *S*.* mutans* PTCC 1683, *A*.* actinomycetemcomitans* ATCC 33384, *P*.* gingivalis* ATCC 33227, and *P*.* intermedia* ATCC 25671. In this way, threshold cycle values (CT) were applied to measuring absolute bacterial concentration from each oral sample and reported as quantity of copy number of each bacterium per sample.^23^

### 2.5 Inflammatory cytokine measurements

Human serum inflammatory cytokine levels of Interleukin‐1 (IL‐1β), Interleukin‐6 (IL‐6), and tumor necrosis factor‐α (TNF‐α) were quantified using specific sandwich enzyme‐linked immunosorbent assays (ELISA). ELISA were performed with the Human kit (R&D, Bio‐Techne kit) based on the procedures recommended by the manufacturer. All collected sera from both groups were assayed on duplicate and the yielded concentrations were expressed as pg/mL, according to calibration curves prepared with cytokine standards contained in the kits. The fluorescence intensity of ELISA plates was measured at 450 nm absorbance.

### 2.6 Statistical analysis

All statistical analysis were performed using SPSS‐22 (SPSS incorporate) at a two‐tailed *p* value of ≤0.05. Normal distribution of data was determined using the *Shapiro–Wilk's* test. Mann–Whitney *U* test was used to define between‐group differences of the three cytokines. A chi‐squared (*X* ^2^) test was also used to assess the differences of demographic characteristics between the groups. Moreover, independent samples *t*‐tests were applied to evaluate significance between the groups for the microbiome bacteria. Effect size statistics including Cohen's *d* (2 × $\frac{t}{\sqrt{n1+n2‐2}}$) and *r* ($\frac{Z}{\sqrt{n}}$) were calculated for the *t*‐test and Mann–Whitney *U* test, respectively, presenting 0.1 (small), 0.3 (medium), and 0.5 (large).^24^ We also used Spearman's rank correlation coefficient (*ρ*) to determine if there was a relationship between the number of pathogenic bacteria in the oral microbiome and the concentration of cytokines.

## 3 RESULTS

### 3.1 3–1 Subjects

The current study included a total of 30 participants distributed in the following groups: AD group (*n* = 15) and healthy group (*n* = 15). The AD group included 8 (53.3%) male and 7 (46.7%) female which were diagnosed with the disease. In interpreting the demographic information, different characteristics of the participants and their relationship with AD were analyzed. Table 2 shows the details of the differences between the control and AD groups. The mean age of participants in the control group was 64.33 ± 3.73, but in the AD group the mean age of patients was 69.47 ± 6.88. The age of the subjects in the AD group was higher than the control group. Statistical analysis showed that there was a significant difference between the control and AD groups in terms of age (*p* < 0.01). The incidence of diabetes, hypertension, and hyperlipidemia was significantly higher in the AD group than in healthy individuals. In addition, level of physical activity and education were two of the most influential characteristics in AD, which were lower in the AD group than control group. It was also found that more than half of the people in the AD group had a family history of the disease. All participants in both groups were married. The results also confirmed that there was a significant difference between the two groups in terms of hypertension, diabetes, hyperlipidemia, family history, education status, and level of physical activity (Table 2). On the other hand, there was no significant difference between BMI, sex, smoking, dentistry services, and occupation between AD and control groups (*p* > 0.05).

**TABLE 2:** Comparison of frequency and significance level of different characteristics between AD and control groups

Characteristics	Healthy group (*n* = 15)	AD group (*n* = 15)	*p* value
Mean ± SD			
Age	64.33 ± 3.73	69.47 ± 6.88	0.019
BMI	25.88 ± 3.26	28.44 ± 5.66	0.147
Frequency (%)			
Hypertension	4 (26.7)	11 (73.3)	0.027
Diabetes	4 (26.7)	11 (73.3)	0.027
Hyperlipidemia	4 (26.7)	10 (66.7)	0.028
Sex			
Male	8 (53.3)	8 (53.3)	>0.5
Female	7 (46.7)	7 (46.7)	
Family history	0 (0.0)	8 (53.3)	0.001
Smoking			
Smoker	2 (13.3)	2 (13.3)	0.64
Non‐smoker	9 (60.0)	11 (73.3)	
Ex‐smoker	4 (26.7)	2 (13.3)	
Level of education			
Illiterate	0 (0.0)	7 (46.7)	0.009
High school	5 (33.3)	6 (40.0)	
Diploma	7 (46.7)	1 (6.7)	
Associate degree	1 (6.7)	1 (6.7)	
Bachelor's degree	2 (13.3)	0 (0.0)	
Dentistry services			
Low	10 (66.7)	12 (80.0)	0.69
Moderate	3 (20.0)	2 (13.3)	
High	2 (13.3)	1 (6.7)	
Marital status			
Married	15 (100)	15 (100)	–
Bachelor	0 (0.0)	0 (0.0)	
Living area			
Urban	14 (93.3)	9 (60.0)	0.031
Rural	1 (6.7)	6 (40.0)	
Inactive	0 (0.0)	10 (66.7)	<0.001
Sedentary	0 (0.0)	4 (26.7)	
Low	10 (66.7)	1 (6.7)	
Moderate	5 (33.3)	0 (0.0)	
Occupation			
Unemployed	0 (0.0)	1 (6.7)	0.66
Self‐employment	5 (33.3)	3 (20.0)	
Employee	5 (33.3)	5 (33.3)	
Homemaker	5 (33.3)	6 (40.0)	

### 3.2 Bacterial quantification

**FIGURE 2:**
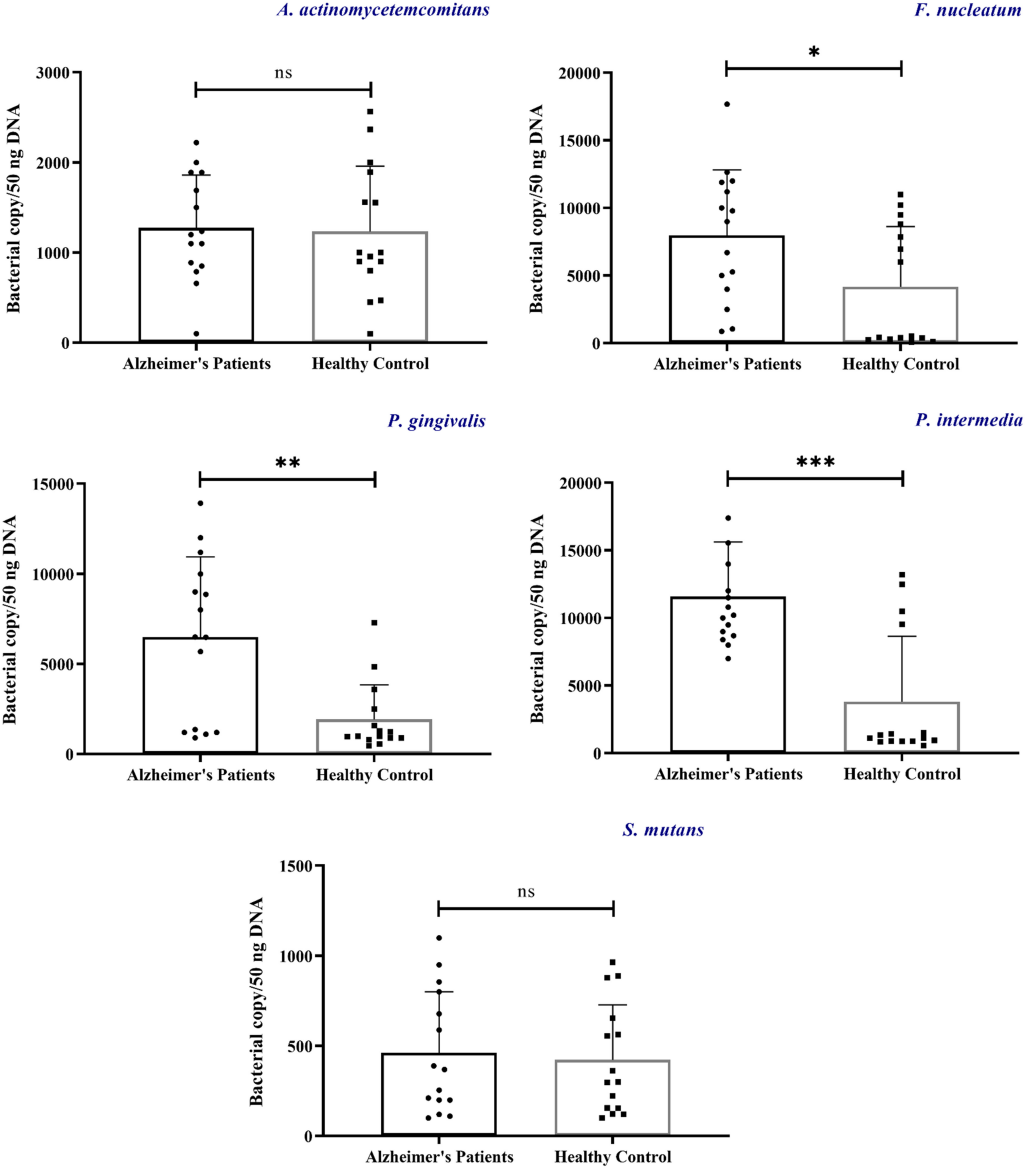
The *t*‐test shows significance difference in the copy number of bacteria in AD and control group. *p < 0.05, **p < 0.01, ***≤0.001

In this case‐control study, qPCR analysis was carried out to compare differences between composition of the most important oral microbiome bacteria in the two groups. Statistical analysis illustrated significant differences in abundance of three bacteria in oral microbiome between AD and healthy groups (Figure 2). Quantification of bacterial genera showed *P*.* gingivalis* was more frequent in the AD compared to the control group (*t* = 3.19, *p* = 0.003, Cohen's *d* = 1.21). The quantity of *F*.* nucleatum* was higher in the oral microbiota of AD group than control group, and there was significant difference in *F*.* nucleatum* abundance between the AD and healthy individuals (*t* = 2.362, *p* = 0.002, Cohen's *d* = 0.89). One other bacterium of the oral microbiome which showed sharply higher abundance in the AD group compared to the healthy group included *P*.* intermedia* (*t* = 3.831, *p* < 0.001, Cohen's *d* = 1.45). Besides that, although *A*.* actinomycetemcomitans* was higher in the AD population, there was no significant difference compared to the healthy individuals (*t* = 1.50, *p* = 0.143). Similarly, even though *S*.* mutans* was relatively frequent in the AD group, there was no meaningful difference in its copy number between two groups (*t* = 1.153, *p* = 0.259).

### 3.3 Cytokine assay

**FIGURE 3:**
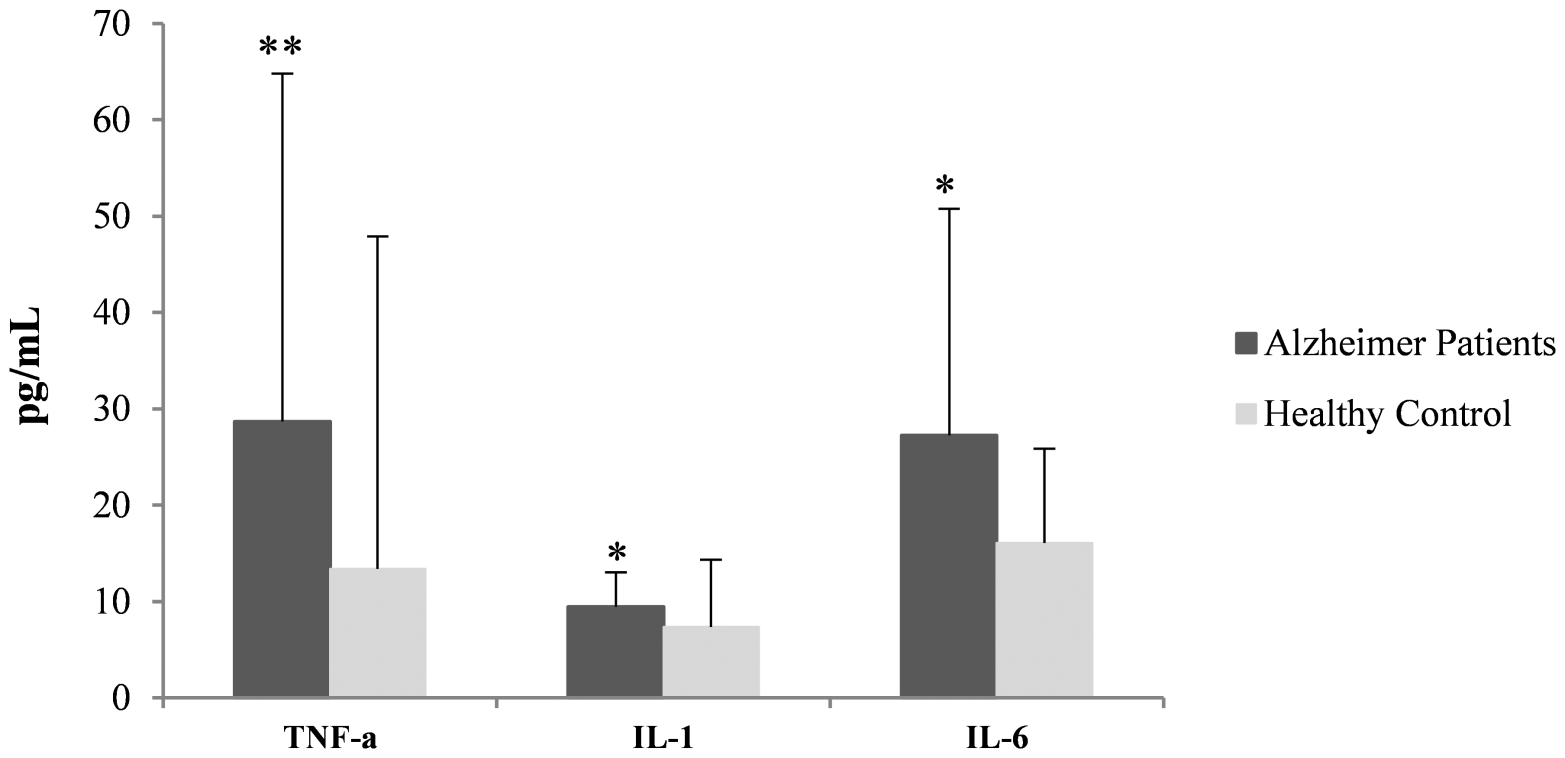
Differences in cytokine concentrations between AD and healthy groups. *Indicates the significant level of difference *p* < 0.05; **Indicates the significant level of difference *p* < 0.001

**FIGURE 4:**
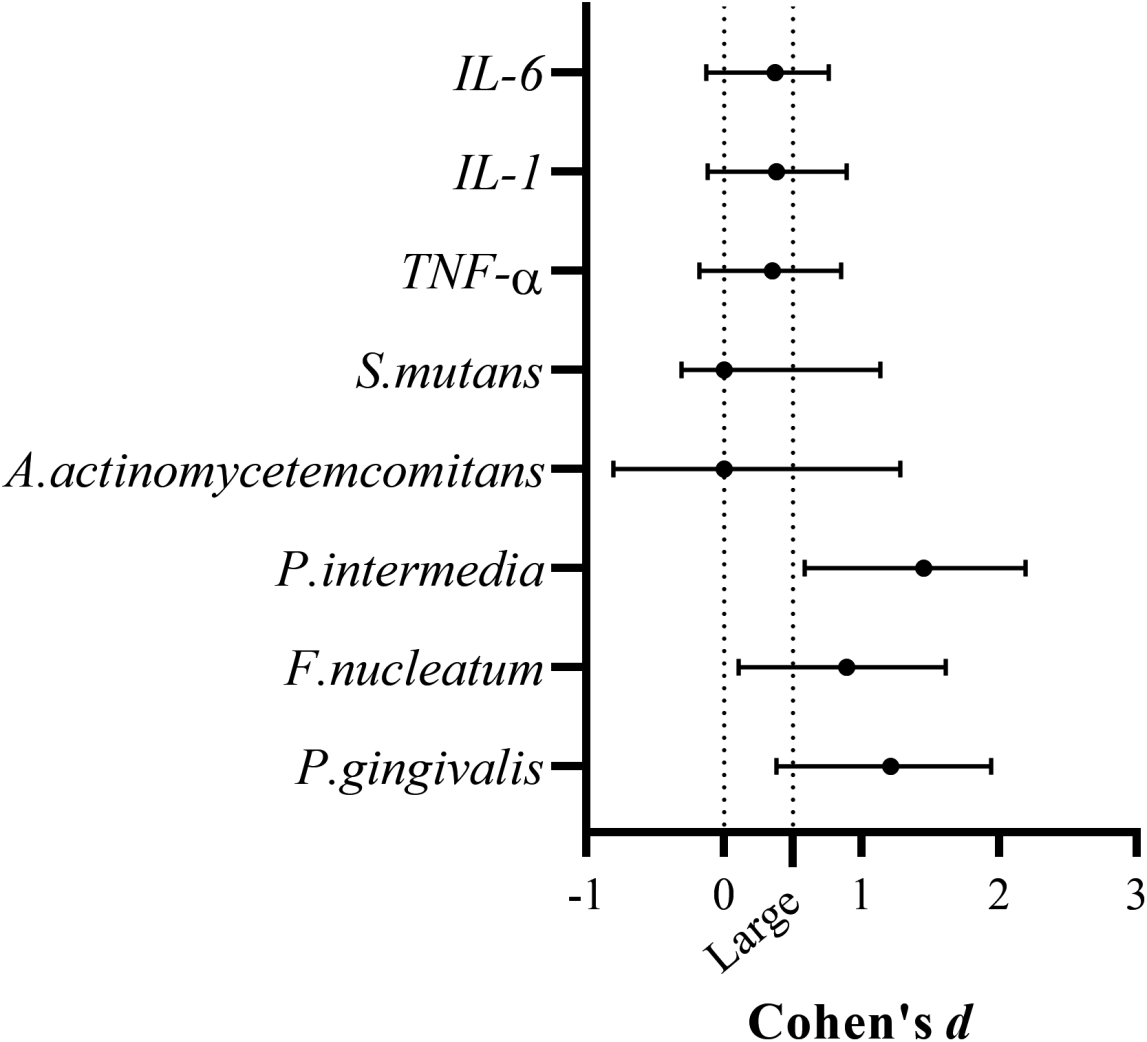
Effect of size estimates for the three cytokines (*r*) and the five studied oral microbiome bacteria (Cohen's *d*) in AD and control group

The ELISA assay results have indicated that the amounts of all three cytokines were higher in AD as compared to the healthy subjects (Figures 3 and 4). Comparison of IL‐1 concentration in the two groups showed that the concentration of this cytokine in the AD group was significantly different compared to the control group (*U* = 62.5, *p* = 0.038). On the other hand, the level of IL‐6 in the AD group was higher than healthy individuals, which was a significant difference in concentration between the two groups (*U* = 64, *p* = 0.044). Not only the concentration of TNF‐α in the AD group was higher than the control group, but also statistical analysis shows a significant level for this concentration difference between the two groups (*U* = 26, *p* < 0.001).

### 3.4 Correlation between bacterial load and cytokines

**FIGURE 5:**
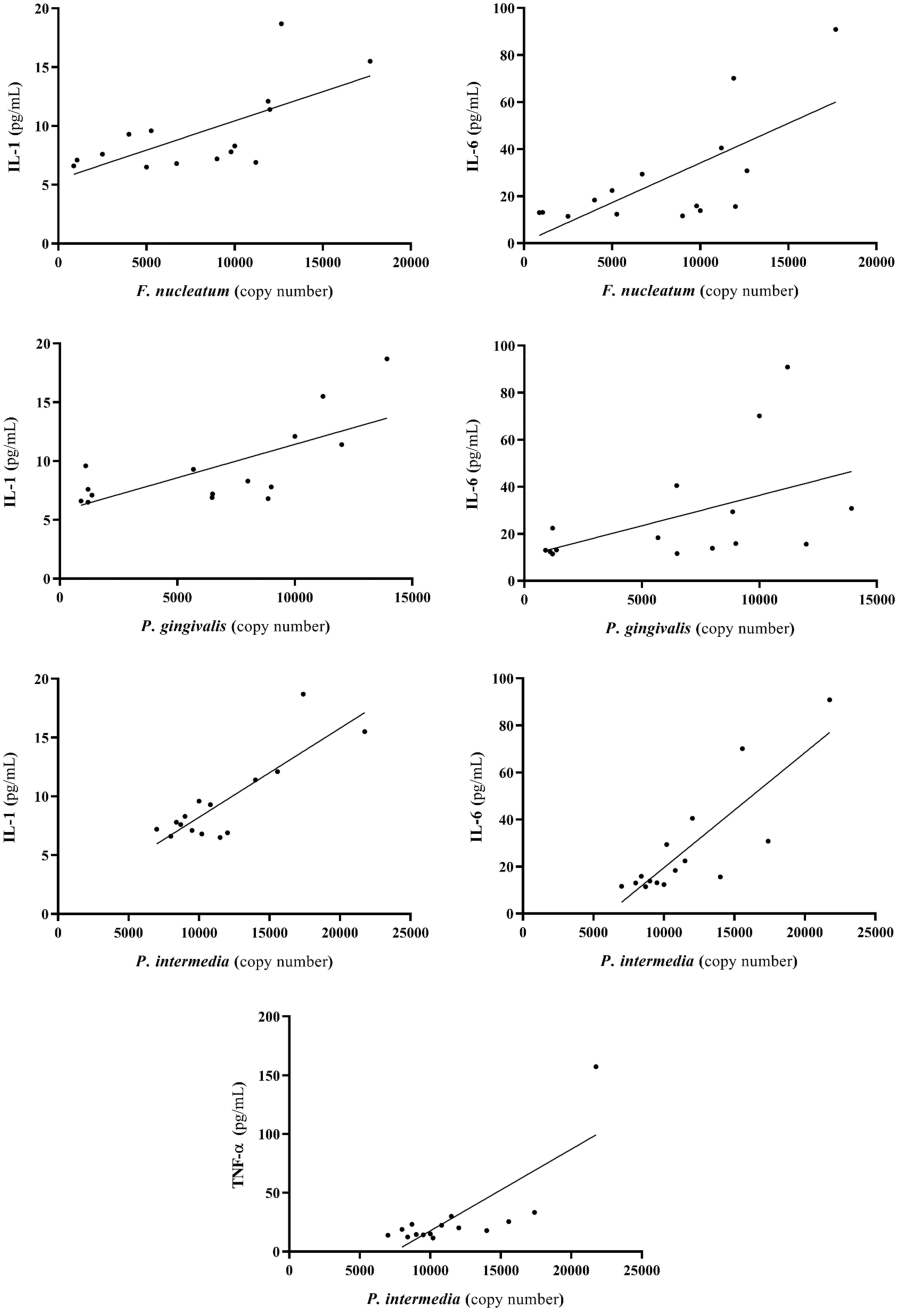
Correlation between copy number of each oral microbiome bacterium and the three inflammatory cytokines

Statistical analysis of the correlation between increasing the number of bacterial microbiome and increasing the concentration of cytokines in the blood of patients showed that there was a significant relationship in this regard (Figure 5). Higher numbers of *P*.* gingivalis* in the oral cavity of AD group showed a significant relationship with increased serum levels of IL‐1 (*ρ* = 0.65, *p* = 0.008) and IL‐6 (*ρ* = 0.60, *p* = 0.018). Similarly, the increase in quantity of *P*.* intermedia* was associated with increased serum concentration of all three cytokines (TNF‐α: *ρ* = 0.67, *p* = 0.006, IL‐1: *ρ* = 0.55, *p* = 0.032, IL‐6: *ρ* = 0.82, *p* < 0.001) in Alzheimer's patients. In addition, higher loads of *F*.* nucleatum* in AD group was associated with increased serum levels of IL‐1 (*ρ* = 0.68, *p* = 0.005) and IL‐6 (*ρ* = 0.64, *p* = 0.009). However, there was no significant relationship between increasing the number of *A*.* actinomycetemcomitan*s and *S*.* mutans* and serum concentrations of the three cytokines in AD group (*p* > 0.05). There was no association between oral bacteria in the control group and increased cytokines in the blood.

## 4 DISCUSSION

Emerging evidence suggested an association between change in human oral microbiota and AD, such that utilization of antibiotics may help to improve AD situation.^18^ In this study, a significant difference in various factors such as hypertension, diabetes, hyperlipidemia, family history, level of education, living area, and level of physical activity was noted between AD and control groups. In line with the results of our study, van Loenhoud et al. reported a significant relationship between AD with age, diabetes, blood pressure, literacy level, and BMI. On the other hand, they stated that there is no significant link between AD with alcohol use, smoking, and hyperlipidemia.^25^ Another study found that although literacy levels were not associated with AD, BMI, smoking, alcohol intake, and hypertension were significantly associated with AD.^26^ Scarmeas et al^27^ reported that although low exercise activity, BMI, ethnicity, age, and literacy levels significantly contributed to AD, smoking and sex did not show any association with it. In another study, it was reported that diabetes, level of education, type of occupation were significantly involved in AD, but no significant difference in BMI and hypertension was observed between Alzheimer's and healthy individuals.^28^ The results of these studies indicate that the underlying factors involved in AD may vary in different geographical areas according to people's lifestyle, ethnicity, and traditions.

In this study, for the first time, we compared the differences and changes of oral microbiome bacteria in healthy and AD groups using the qPCR technique, as well as although there is no study that compared oral microbiome bacteria by direct oral sampling in AD and healthy individuals, studies have shown an increase or decrease antigens of oral microbiota main bacteria in the blood or brain tissue of both groups. Results of present study showed that the frequency of the five oral microbiome bacteria were higher in Alzheimer's patients than in healthy individuals. The difference was significant for *P*.* gingivalis*, *F*.* nucleatum*, and *P*.* intermedia*. Although there have been very few studies looking at changes in the oral microbiota in AD, the limited studies have pointed to a significant association. Our observation was in agreement with findings of Stein et al. who screened serum samples of AD patients for specific IgG antibodies against *P*.* gingivalis*, *A*.* actinomycetemcomitans*, *P*.* intermedia*, and *F*.* nucleatum*. Antibodies against *P*.* gingivalis*, *F*.* nucleatum*, and *P*.* intermedia* were remarkably higher in Alzheimer's patients in comparison with healthy subjects. Also, there was no significant difference between the two groups in regards to *A*.* actinomycetemcomitan*s.^29^ Consistently, a study was conducted to assess interactive correlation between different genera of oral microbiome bacteria and the incidence of AD which reported that there was no correlation between *S*.* mutans* and incidence of AD. However, *F*.* nucleatum* and *P*.* intermedia* were predisposing pathogenic bacteria in the incidence of AD.^30^ Our results are in line with the study conducted by Kamer et al^31^ who compared the levels *P*.* gingivalis* between AD and normal subjects, however, in contrast to our results, they showed *A*.* actinomycetemcomitans*‐specific antibody in Alzheimer's patients were significantly higher than those in healthy individuals. Na et al^32^ reported that the diversities of *Fusobacterium* and *Porphyromonas* genera in the oral cavity, as conducted by genome sequencing, were higher in Alzheimer's patients in comparison with those in healthy individuals. Similarly, according to the results of Noble et al,^33^ high levels of IgG against *P*.* gingivalis* were correlated to cognitive impairment. Similar to our findings, in a pilot study it was reported that *P*.* gingivalis* was a key bacterium associated with cognitive conditions,however, there was no significance difference in terms of *P*.* intermedia* in the two groups. On the other hand, *A*.* actinomycetemcomitans* had no role in progression of AD.^34^ In another study, there was a correlation between major oral bacteria and AD. They noticed presence of *P*.* gingivalis* LPS in the brain of Alzheimer's patients which may have been linked to an inflammatory state.^35^ In accordance with the results of our study, Kamer et al. reported the only shift in *P*.* gingivalis* counts in the AD patients among different oral pathogenic bacteria including *A*.* actinomycetemcomitans*, *T*.* denticola*, *T*.* forsythia*, *P*.* intermedia*, and *P*.* gingivalis*.^18^ It can be stipulated that the increase of pathogenic bacteria in the composition of oral microbiome causes these bacteria to invade the brain. This may be a probable mechanism for etiology of AD. However, it is not yet clear how oral bacteria can gain access to brain and spread. There are some suggested pathways such as direct infection and damage to endothelial cells protecting the blood–brain barrier, infection and spreading through cranial nerves (e.g., olfactory or trigeminal) to the brain, and infection of monocytes followed by brain recruitment. After accessing the brain, bacteria such as *P*.* gingivalis* can spread slowly over many years from neuron to neuron along anatomically connected pathways.^36^

In this study, the inflammatory reactions induced by different cytokines including IL‐1, IL‐6, and TNF‐α in the serum of Iranian AD patients compared to healthy individuals showed that all three cytokines were meaningfully higher in Alzheimer's patients. Similar to the results of our study, Babić Leko et al^37^ reported that TNF‐α, IL‐1, and IL‐6 levels were significantly higher in the CSF of AD group than in the healthy individuals. In another study, it was reported that although serum levels of TNF‐α and IL‐1 in Alzheimer's patients were significantly higher than in healthy individuals, there was no significant difference in IL‐6 between the two groups (Demirci et al^38^). In line with the results of our study, Azzam et al^39^ declared that serum concentrations of IL‐6 and TNF‐α in Alzheimer's patients significantly increased compared to those in healthy individuals. In another study in 2009, it was reported that although serum levels of TNF‐α were significantly higher in Alzheimer's patients, there was no significant increase in IL‐1 and IL‐6 levels in the patients. Finally, it was stipulated that IgG antibodies against periodontal pathogens such as *P*.* gingivalis*, *A*.* actinomycetemcomitans*, and *T*.* forsythia* lead to high levels of TNF‐α in plasma of patients with AD.^31^ In contrast, Lanzrein et al^40^ did not observe a significant difference in serum concentrations of IL‐1, IL‐6, and TNF‐α between patients with AD and healthy individuals. Increased inflammation by cytokines is one of the mechanisms of perturbation of oral microbiome bacteria that lead to nerve and brain damage resulting in AD.^20^ However, the difference between some of the results of our study and other studies may be due to differences in sample size.

## 5 CONCLUSION

These results suggest that an increase in the number of some oral pathogenic bacteria and consequently an increase in systemic inflammation may be one of the factors associated with AD and may contribute to a better clinical diagnosis of AD in the future. These initial observations require further extensive studies to evaluate more extensive genera of oral bacteria, their virulence factors, and possible diffusion of biological products into brain and nerve tissue. Finally, due to the inability of existing medical or psychological treatments for AD, the discovery of changes in the oral microbiome and cytokines involved in systemic inflammation, as new modulators and mediators of AD, could lead to the development of new preventive and therapeutic strategies.

## AUTHOR CONTRIBUTIONS

Majid Taati Moghadam and Aref Shariati developed the idea and drafted the manuscript. Nour Amirmozafari, Ali Mojtahedi, Babak Bakhshayesh, and Faramarz Masjedian Jazi co‐wrote, developed, and edited the manuscript.

## CONFLICT OF INTEREST

The authors declare no conflicts of interest relevant to this article.

## Data Availability

The data that support the findings of this study are available from the corresponding author upon reasonable request.
